# P-2288. Antibiotic Use and Infectious Complications in Hematological Patients Undergoing CAR T-Cell Therapy

**DOI:** 10.1093/ofid/ofae631.2441

**Published:** 2025-01-29

**Authors:** Pedro Puerta-Alcalde, Maria Calbacho, Rebeca Bailen, Ana Africa Martin, Inmaculada Garcia, Borja Puertas, Anna Vilalta, Mercedes Montoro, Sergi Anguera, Valentin Ortiz, Carlos Fernandez-Larrea, Alex Soriano, Carolina Garcia-Vidal

**Affiliations:** Hospital Clinic de Barcelona, Barcelona, Catalonia, Spain; Hospital Universitario 12 de Octubre, Madrid, Madrid, Spain; Hospital Universitario Gregorio Marañon, Madrid, Madrid, Spain; Hospital Universitario de Salamanca, Salamanca, Castilla y Leon, Spain; Hospital Universitario de Salamanca, Salamanca, Castilla y Leon, Spain; Hospital Universitario de Salamanca, Salamanca, Castilla y Leon, Spain; Hospital Clinic de Barcelona, Barcelona, Catalonia, Spain; Hospital Clinic de Barcelona, Barcelona, Catalonia, Spain; Hospital Clinic de Barcelona, Barcelona, Catalonia, Spain; Hospital Clinic de Barcelona, Barcelona, Catalonia, Spain; Hospital Clinic de Barcelona, Barcelona, Catalonia, Spain; Hospital Clínic de Barcelona, Barcelona, Catalonia, Spain; Hospital Clinic, Department of Hematology, Barcelona, Spain, Barcelona, Catalonia, Spain

## Abstract

**Background:**

Patients undergoing chimeric antigen receptor (CAR) T-cell therapy face significant infection risks. Current data on the frequency and nature of these infections remain limited.

Table 1

**Figure:**
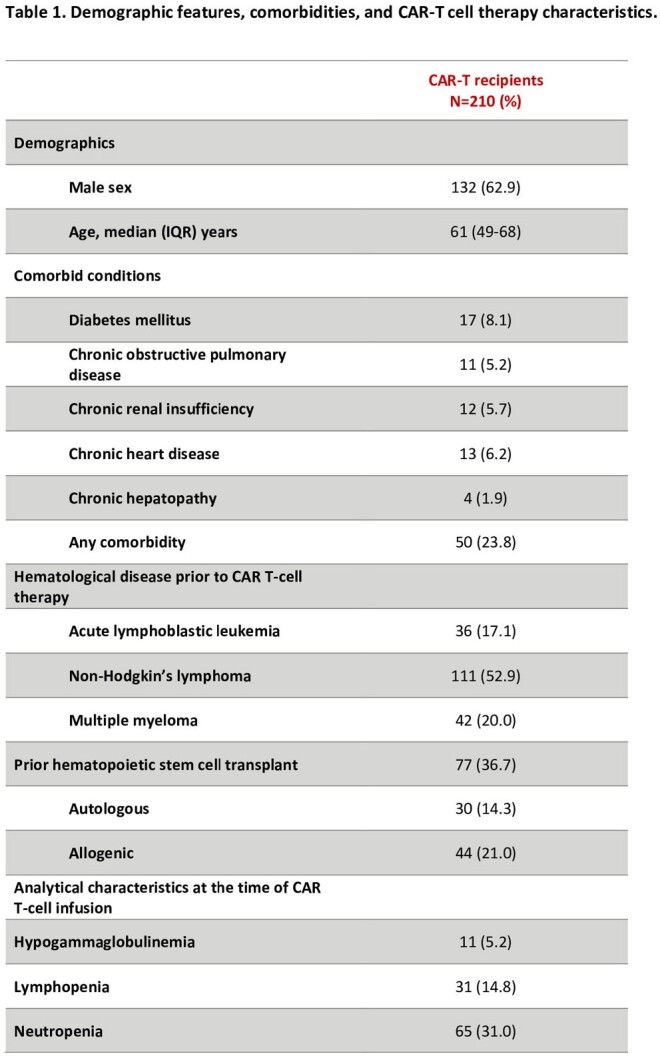

**Methods:**

Prospective multicenter cohort including all consecutive patients treated with CD19 or BCMA CAR T-cell therapy from January 2022 to February 2024 in three university hospitals in Spain. All participants provided informed consent and were monitored for 90 days post-treatment.

Table 1 (continued)

**Figure:**
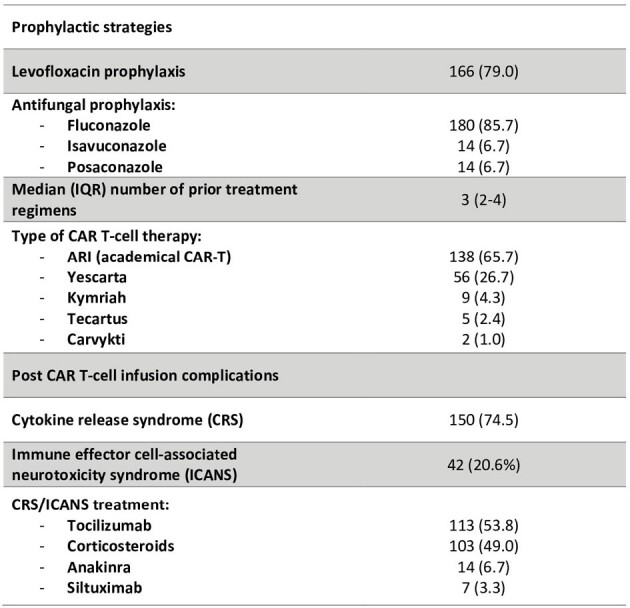

**Results:**

A total of 210 patients were enrolled. Demographics, comorbidities, and specific therapy details are outlined in Table 1. Overall, 71% of patients experienced fever at a median of 2 days (IQR 1-3) after CAR T-cell infusion. Antibiotics were administered to 79.3% of patients for a median duration of 7 days (IQR 4-11) with meropenem (57.6%) and piperacillin-tazobactam (40.5%) being the most frequently used. Bacteremia was evidenced in 17 (8.1%) patients, with 6 (2.9%) patients experiencing multiple episodes. The median onset of first bacteremia was 27 days (IQR 16.5-36), with 14/17 (82.3%) episodes occurring over 2 weeks after CAR T-cell infusion. Predominant pathogens were *P. aeruginosa* (5, 2.4%), coagulase-negative staphylococci (5, 2.4%), and *E. coli* (4, 1.9%). *Clostridioides difficile* infection occurred in 7 (3.3%) patients. Viral infections were reported in 37 (17.6%) patients, primarily due to SARS-CoV-2 (22, 10.5%) and Influenza (6, 2.9%), while fungal infections were noted in 10 (4.8%) patients (4 aspergillosis, 3 candidemia, 1 mucormycosis, 1 other mold, and 1 possible fungal infection). ICU admission was required for 38 (18.1%) patients, with infections prompting 6 of these cases. The 90-day mortality rate stood at 6.2%, with 61.5% of these deaths attributed to infections. Patients suffering any infection (bacterial, viral, or fungal) had significantly higher 90-day mortality (17.2% vs 2.0%, p < 0.001). Table 2 shows the different 90-day mortality rates regarding the type of infection.

**Figure:**
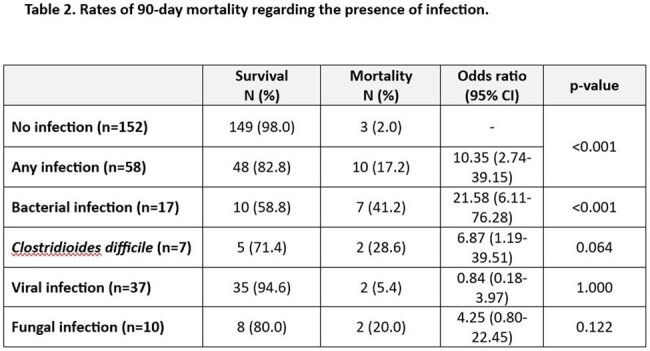

**Conclusion:**

Antibiotic use is prevalent immediately following CAR T-cell infusion, despite a low initial bacterial infection rate. Nonetheless, infections within the first 90 days post-CAR-T infusion are common and correlate with worse outcomes.

**Disclosures:**

All Authors: No reported disclosures

